# E1021K mutation in PIK3CD gene: clinical heterogeneity and therapeutic implications in three pediatric APDS cases

**DOI:** 10.1515/biol-2025-1268

**Published:** 2026-03-10

**Authors:** Changxiao Li, Linlin Han, Qian Li

**Affiliations:** Department of Respiratory Interventions, Affiliated Children’s Hospital, Shandong University, Jinan City, China

**Keywords:** immunodeficiency 14, PIK3CD, activated PI3K-δ syndrome, APDS, lymphonodular hyperplasia

## Abstract

The aim of this study was to characterize the clinical manifestations, treatment responses, and prognostic indicators of activated PI3K-δ syndrome (APDS) in pediatric patients. Clinical data from three patients diagnosed with APDS in our department were retrospectively analyzed. All patients carried the same heterozygous E1021K (c.3061G > A) gain-of-function mutation in the PIK3CD gene. Immunoglobulin levels varied: IgM was normal or elevated, while IgG and IgA were normal or decreased, with the extent of change correlating with disease severity. All three children received anti-infective therapy, resulting in significant improvement in clinical symptoms and chest imaging findings. Bronchoscopic re-evaluation in Cases 1 and 2 showed marked regression of airway mucosal hyperplasia. Following diagnosis, Cases 1 and 2 received regular immunoglobulin replacement therapy, which reduced the frequency of infections. Case 2 was treated with rapamycin as targeted therapy, leading to significant improvement in hepatosplenomegaly. In conclusion, bronchoscopic detection of nodular lymphoid hyperplasia is a diagnostic hallmark of APDS. Progressive T-cell exhaustion and immunoglobulin dysregulation may serve as biomarkers of disease severity. Targeted therapy, such as rapamycin, demonstrates clinical efficacy. This case series underscores the variable expressivity of APDS even among individuals sharing the same pathogenic variant, emphasizing the need for personalized management.

## 1 Introduction

Activated PI3K-δ syndrome (APDS), also known as immunodeficiency 14 (OMIM:615513), is a rare autosomal dominant primary immunodeficiency caused by gain-of-function mutations in PIK3CD. Since its initial description by Angulo et al. in 2013 [1], over 100 cases have been reported worldwide. In China, 58 cases have been documented, primarily as larger cohort studies or sporadic reports [2]. Among the known PIK3CD gain-of-function mutations, the E1021K (c.3061G > A) variant is the most frequently reported globally [3], 4]. However, the phenotypic spectrum and long-term management of pediatric APDS, especially within the Chinese population, remain incompletely characterized. This article presents the clinical features, laboratory findings, and treatment outcomes of three children admitted to our department with recurrent respiratory infections, lymphadenopathy, and bronchoscopy-confirmed nodular lymphoid hyperplasia. We aim to enhance awareness of this disease among pediatricians to facilitate early recognition, timely diagnosis, and appropriate intervention.

## 2 Case information

**Case 1**: A 3-year-and-11-month-old girl was admitted to our department in March 2017 with a history of recurrent cough with purulent sputum for over one year and fever for one day. In the preceding year, she had been hospitalized four times for severe pneumonia, with each episode lasting approximately 7–13 days; symptoms recurred shortly after each improvement. During this period, she underwent two bronchoscopic examinations (8 months apart), both of which indicated bronchial mucosal inflammation and nodular lymphoid hyperplasia. Pathological examination of two mucosal biopsy specimens revealed chronic inflammatory changes. Contrast-enhanced chest CT showed consolidation in the right lower lobe and enlarged right hilar lymph nodes. Autoantibody panels, immunoglobulin levels, and complement levels were all within normal ranges. The patient had experienced two episodes of otitis media after the age of 3. Her mother had a normal pregnancy; this was her first pregnancy, resulting in a full-term cesarean delivery of twins. The patient was the younger twin, with a healthy older twin sister. Both parents were healthy, non-consanguineous, and had no relevant family history.

Physical examination: Weight was 16 kg. She was conscious and moderately nourished. Multiple lymph nodes, approximately soybean-sized, were palpable in the bilateral cervical, axillary, and inguinal regions. No scars were present. Breathing was stable, with coarse breath sounds and scattered crackles audible in both lungs. The liver and spleen were not palpable below the costal margin. No digital clubbing was observed. Examinations of other systems were unremarkable.

**Figure 1:**
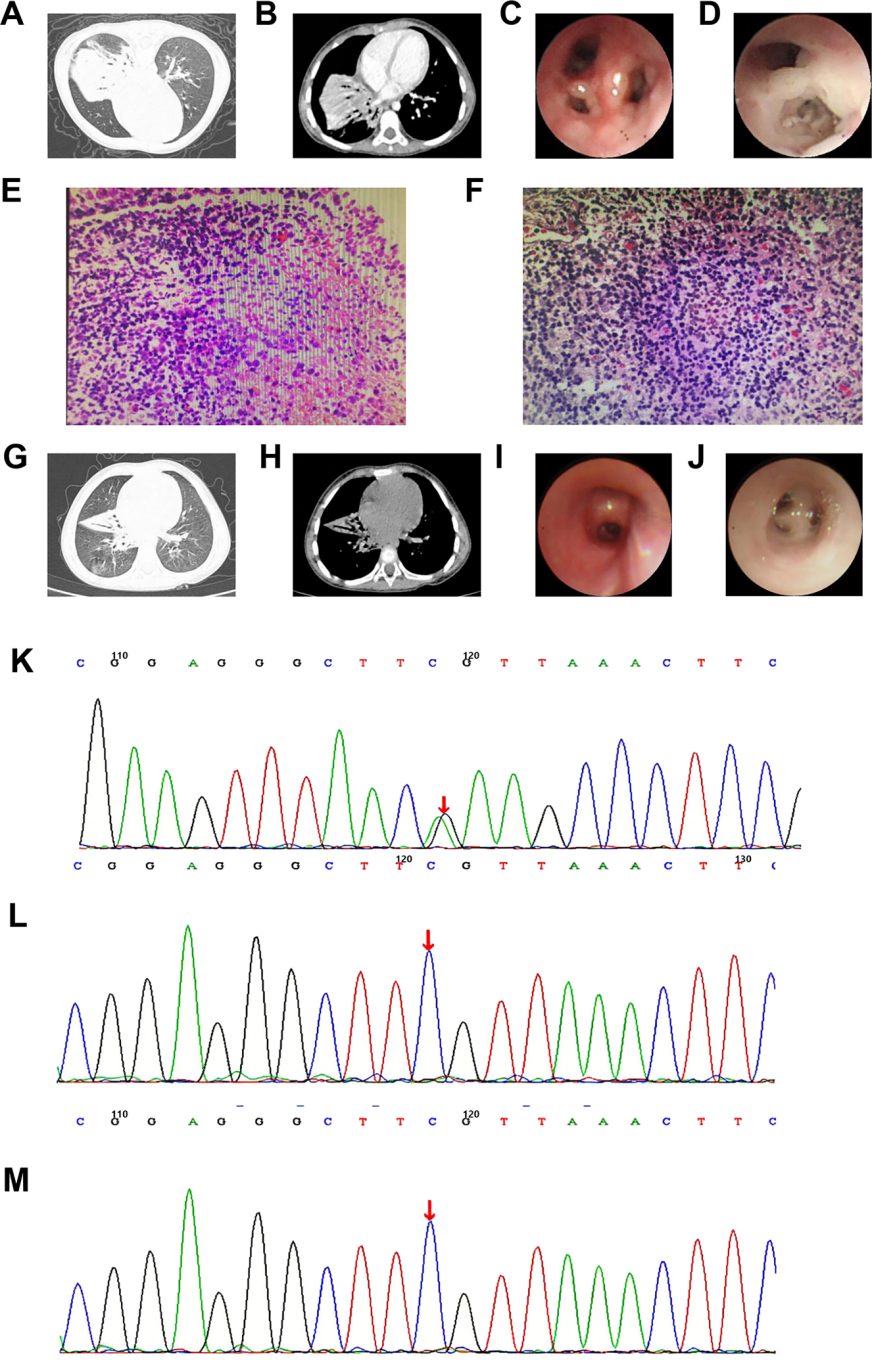
Examinations findings of case 1. (A) Initial Lung window on chest CT before treatment. (B) Initial chest CT scan of mediastinal window before treatment. (C) Bronchoscopy revealed significant bronchial mucosal follicular hyperplasia before treatment. (D) Bronchoscopy revealed abundant yellow-white secretions obstructing the airway before treatment. (E) Mucosal biopsy (HE × 40) revealed numerous lymphocytes infiltrating and forming focal aggregations. (F) Lymph node biopsy (HE × 40) showed reactive lymphoid hyperplasia. (G) Follow-up chest CT of lung window three weeks after treatment. (H) Follow-up chest CT of mediastinal window three weeks after treatment. (I) Bronchoscopy showed the lymphoid follicles were basically disappeared after treatment. (J) Bronchoscopy revealed a significant reduction in secretions after treatment. (K) The patient carried a heterozygous mutation c.3061G > A (p.E1021K) in the PIK3CD gene. (L) No variation seen in the father of the child. (M) No variation seen in the mother of the child.

Auxiliary examinations: Complete blood count: WBC 6.65 × 10^9^/L, lymphocyte percent 35.00 %, RBC 3.99 × 10^12^/L, Hb 111.00 g/L, platelets 186.00 × 10^9^/L. Sputum culture showed normal respiratory flora. Tests for respiratory viruses, T-SPOT, EBV antibodies, and CMV antibodies were all negative. The PPD test was negative. Immunoglobulin levels and T-lymphocyte subset values are shown in Table 1. Lymph node ultrasonography revealed enlarged lymph nodes with normal internal structure in the bilateral neck, axillae, and inguinal regions. Abdominal ultrasound indicated mesenteric lymphadenopathy. Contrast-enhanced chest CT was consistent with pneumonia, showing atelectasis of the right middle and lower lobes with features suggestive of local ischemic necrosis; multiple enlarged lymph nodes were noted in the right hilum and mediastinum (Figure 1A and B).

**Table 1:** Specific immunological indexes of 3 cases.

		IgM (g/L)	IgA (g/L)	IgG (g/L)	Total T-cell	CD4+ T lymphocytes %	CD8 + T lymphocytes %	CD4/CD8 ratio
Case 1	Reference range	1.10–1.80	0.58–1.00	6.60–10.39	62–80	35–51	22–38	1.0–2.1
	Time							
	Initial hospitalization	1.96	0.80	5.93				
	6 months later	1.65	0.83	9.14				
	1 year later	2.19	0.74	7.79	75.28	25.18	49.35	0.51
Case 2	Reference range	1.20–2.26	0.85–1.71	7.91–13.07	66–76	33–41	27–35	1.1–1.4
	Time							
	Initial hospitalization	7.35	0.058	10.20				
	1 month later	7.43	<0.06	10.20	78.67	26.64	49.71	0.54
	2 months later	8.27	0.06	10.00	76.22	18.31	75.09	0.24
Case 3	Reference range	1.2–2.26	0.85–1.71	7.91–13.07	66–76	33–41	27–35	1.1–1.4
	Time							
	1 year ago	2.14	0.666	8.51	73.98	37.58	35.12	1.07
	Initial hospitalization	2.30	1.47	8.77	48.61	18.57	26.89	0.69

**Figure 2:**
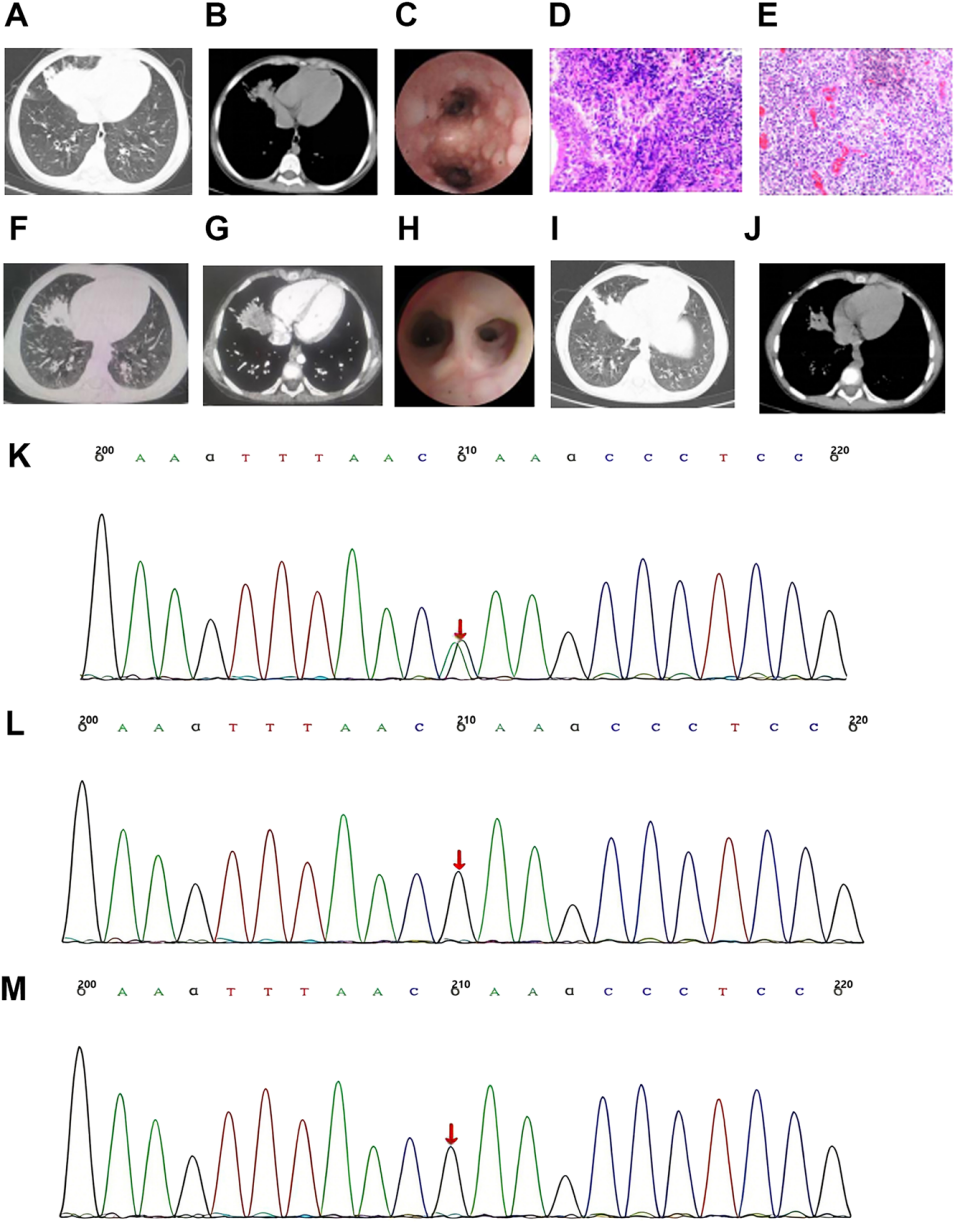
Examinations findings of case 2. (A) Initial Lung window on chest CT before treatment. (B) Initial chest CT scan of mediastinal window before treatment. (C) Significant bronchial mucosal follicular hyperplasia observed under bronchoscopy before treatment. (D) Mucosal biopsy (HE × 40): large amount of lymphocytes aggregated into lymphoid follicle-like structures. (E) Lymph node biopsy (HE × 40) showed non-specific lymphadenitis. (F) Follow-up chest CT with pulmonary window after 2 weeks of treatment. (G) Follow-up chest CT with mediastinal window after two-week treatment. (H) The lymphoid follicles are essentially disappeared upon bronchoscopy after treatment. (I) Follow-up chest CT scan with lung window 1 month later. (J) Follow-up thoracic CT with mediastinal window 1 month later. (K) The affected child PIK3CD c.3061G > A (p.E1021K) heterozygous mutation in gene. (L) No mutation detected in the father of the affected child. (M) No mutation detected in the mother of the affected child.

Treatment: Bronchoscopy was performed the day after admission, revealing bilateral bronchial mucosal inflammation with follicular hyperplasia, particularly in the right middle and lower lobes. Mucosal biopsies taken from these areas showed edematous and narrowed bronchial sub-segments with gray-white fibrinous secretions (Figure 1C and D). Pathological examination demonstrated dilated and congested subepithelial vessels with focal hemorrhage, alongside extensive lymphocytic infiltration forming focal aggregates mixed with few plasma cells (Figure 1E). A left inguinal lymph node biopsy performed on day 5 showed reactive hyperplasia (Figure 2F). Repeat bronchoscopy on day 12 yielded similar findings, with mucosal biopsies consistent with chronic bronchitis. The patient developed a right pneumothorax post-procedure, which was managed with thoracentesis and closed drainage. Anti-infective therapy with cefoperazone-sulbactam was initiated. Culture of bronchoalveolar lavage fluid yielded *Streptococcus pneumoniae*. Due to rising inflammatory markers, linezolid was added for 10 days. The patient’s clinical symptoms improved following treatment. Follow-up chest CT (Figure 1G and H) showed resolution of the consolidation with residual atelectasis in the right middle lobe. Enlarged lymph nodes persisted in the right pulmonary hilum, behind the superior vena cava, and below the carina; however, repeated bronchoscopy showed resolution of the follicular hyperplasia (Figure 1I and J). The patient was subsequently discharged on medication.

Genetic testing: Genetic analysis (Beijing Maijino Medical Testing Institute, sample number: 17C003006) revealed a heterozygous missense mutation in the PIK3CD gene: c.3061G > A (p.Glu1021Lys, E1021K). This mutation was not detected in either parent (Figure 1K–M).

Follow-up: Following discharge, the patient experienced intermittent cough with purulent sputum. Within the first year, she was hospitalized 4–5 times for pneumonia. Investigations were positive for EBV, CMV, and adenovirus antibodies, and sputum cultures grew *S. pneumoniae* and Salmonella. One year post-discharge, she began regular immunoglobulin replacement therapy (initially 10 g every 2 months, increased to 12.5 g monthly since 2019), which reduced the frequency of infections. Physical examination revealed mild hepatosplenomegaly and progressively enlarging inguinal lymph nodes (largest measuring 3 cm), which regressed after anti-infective treatment. Follow-up imaging two years later indicated the development of bronchiectasis.

**Case 2:** A 7-year-and-2-month-old girl was admitted to our department in March 2017 with a history of recurrent cough for over two months. The cough was continuous, occurring both day and night, non-paroxysmal, and not associated with fever or wheezing. Self-administered oral and intravenous medications had been ineffective. Chest CT revealed: (1) bilateral infectious lesions in the lungs; (2) multiple enlarged lymph nodes in the mediastinum and bilateral axillae. Bronchoscopy showed a rough mucosal surface with granular protrusions. The admission diagnosis was “cough of unknown etiology.” Since birth, the patient had a history of recurrent otitis media. Before the age of 3, she was hospitalized approximately 7 times per year for pneumonia. Between ages 3 and 5, she was treated for pneumonia about 4 times annually, and at age 6, she was hospitalized once for pneumonia, with full recovery each time. Both parents were healthy, non-consanguineous, and had no relevant family history.

Physical examination: Weight was 23 kg. She was conscious and well-nourished, with facial erythema noted. Multiple enlarged lymph nodes, measuring approximately 0.5 × 0.5 cm, were palpable in both axillae. They were soft, mobile, non-tender, and not adherent to surrounding tissues. Respiration was steady with coarse breath sounds bilaterally. The liver was not enlarged, but the spleen was palpable 2 cm below the costal margin. No digital clubbing was observed. Examinations of other systems were unremarkable.

Ancillary tests: Complete blood count: WBC 6.18 × 10^9^/L, lymphocyte percent: 26.00 %, hemoglobin 95.00 g/L, platelets 140.00 × 10^9^/L. Serology was positive for adenovirus and respiratory syncytial virus IgM antibodies, while EBV antibodies, EBV-DNA, CMV antibodies, and CMV-DNA were negative. TB infection T-cell test and PPD test were negative. Immunological profiles are shown in Table 1. Biochemistry indicated alanine aminotransferase (ALT) 72 U/L, aspartate aminotransferase (AST) 62 U/L, and alkaline phosphatase (ALP) 646 U/L; remaining indicators were normal. Abdominal ultrasound showed: (1) multiple enlarged intra-abdominal lymph nodes; (2) heterogeneous liver parenchymal echotexture with thickened Glisson’s capsule; (3) splenomegaly (spleen tip 2.7 cm below the left costal margin). Lymph node ultrasound confirmed enlargement of bilateral cervical, axillary, and inguinal nodes. Chest CT demonstrated pneumonia with consolidation in the right middle lobe, along with multiple enlarged axillary lymph nodes (Figure 2A and B).

Treatment: The patient received meropenem for infection. Bronchoscopy was performed the day after admission, revealing tracheobronchial mucosal inflammation with extensive follicular hyperplasia in the lower trachea and both main bronchi (Figure 2C). Mucosal biopsies were taken from the left main bronchus and right middle lobe bronchus, along with a transbronchial lung biopsy from the right middle lobe. Histopathology of the bronchial biopsies showed numerous subepithelial lymphoid aggregates forming follicle-like structures, accompanied by perifollicular fibrosis and mucus gland hyperplasia (Figure 2D). Lung tissue biopsy showed dilated and congested vessels with epithelial denudation. A cervical lymph node biopsy revealed paracortical hyperplasia with sinus histiocytosis and prominent B-cell hyperplasia, consistent with acute non-specific lymphadenitis (Figure 2E). Immunohistochemistry was positive for CD30, CD20, CD3, CD21, CD79α, CD4, CD8, CD68, and MPO; and negative for EMA, ALK, and CD10. EBV-DNA was detected in the lymph node tissue at 1.39 × 10^4^ copies/mL, and IgM antibodies against herpes simplex virus were elevated at 11.31 AU/mL. Consequently, acyclovir was added for antiviral therapy. Follow-up chest CT (Figure 2F and G) after treatment showed improvement in the pulmonary lesions and revealed localized emphysematous changes in both lower lobes. Repeat bronchoscopy 17 days later (Figure 2H) showed persistent tracheobronchial mucosal inflammation with pale mucosa and slight roughening at the orifice of the right middle lobe bronchus, but a marked reduction in follicular hyperplasia compared to the previous exam. Follow-up ultrasonography showed decreased cervical lymph node size and mild reduction in spleen size. The patient’s condition improved, and she was discharged.

Genetic testing: Genetic analysis (Beijing Maikenon Medical Laboratory, sample number: 17C003058) identified a heterozygous mutation in the PIK3CD gene: c.3061G > A, resulting in an amino acid change p.Glu1021Lys (E1021K). This *de novo* missense mutation was not detected in either parent (Figure 2K–M).

Follow-up: The patient was re-admitted one month after discharge due to severe pneumonia and treated with meropenem. Testing was positive for *Mycoplasma pneumoniae* IgM and cytomegalovirus IgM antibodies. Updated immunological profiles are shown in Table 1. Follow-up chest and sinus CT (Figure 2I and J) indicated: (1) progression of pulmonary inflammation compared to prior imaging, with partial bronchiectasis in both lungs and persistent localized emphysematous changes; (2) sinusitis and bilateral mastoiditis. Her symptoms improved with treatment. Subsequent abdominal ultrasound revealed hepatomegaly, thickened Glisson’s capsule, splenomegaly, biliary sludge, a widened common bile duct, multiple abdominal lymph nodes, and diffuse colonic wall thickening. During follow-up in our clinic, she received monthly immunoglobulin infusions and oral clarithromycin (1 mg/kg/day), with regular therapeutic drug monitoring initiated one and a half years ago. On this regimen, her liver, spleen, and lymph nodes have decreased in size, and her respiratory infections have been well-controlled.

**Case 3:** A 7-year-old boy was admitted to our department in December 2019 for a 3-day history of cough and throat phlegm. Outpatient chest CT revealed sinusitis, bilateral otomastoiditis, pneumonia, and partial opacification of the right middle lobe. At 3 months of age, he was diagnosed with immune thrombocytopenic purpura and had a positive cytomegalovirus test; this condition recurred 6–7 times during spring and autumn until the age of 5, with no recurrence thereafter. From age 5 onward, he experienced repeated episodes of pneumonia. In the six months prior to admission, he was hospitalized for “pneumonia” every 1–2 months. Both parents were healthy, non-consanguineous, and had no relevant family history.

Physical examination: Weight was 22 kg. He was conscious and moderately nourished. Multiple lymph nodes, approximately soybean-sized, were palpable in the bilateral cervical, axillary, and inguinal regions. Breathing was steady, with coarse breath sounds, occasional fine moist rales, and scattered wheezing heard in both lungs. The liver and spleen were not palpable below the costal margin. No digital clubbing was observed. Examinations of other systems were unremarkable.

**Figure 3:**
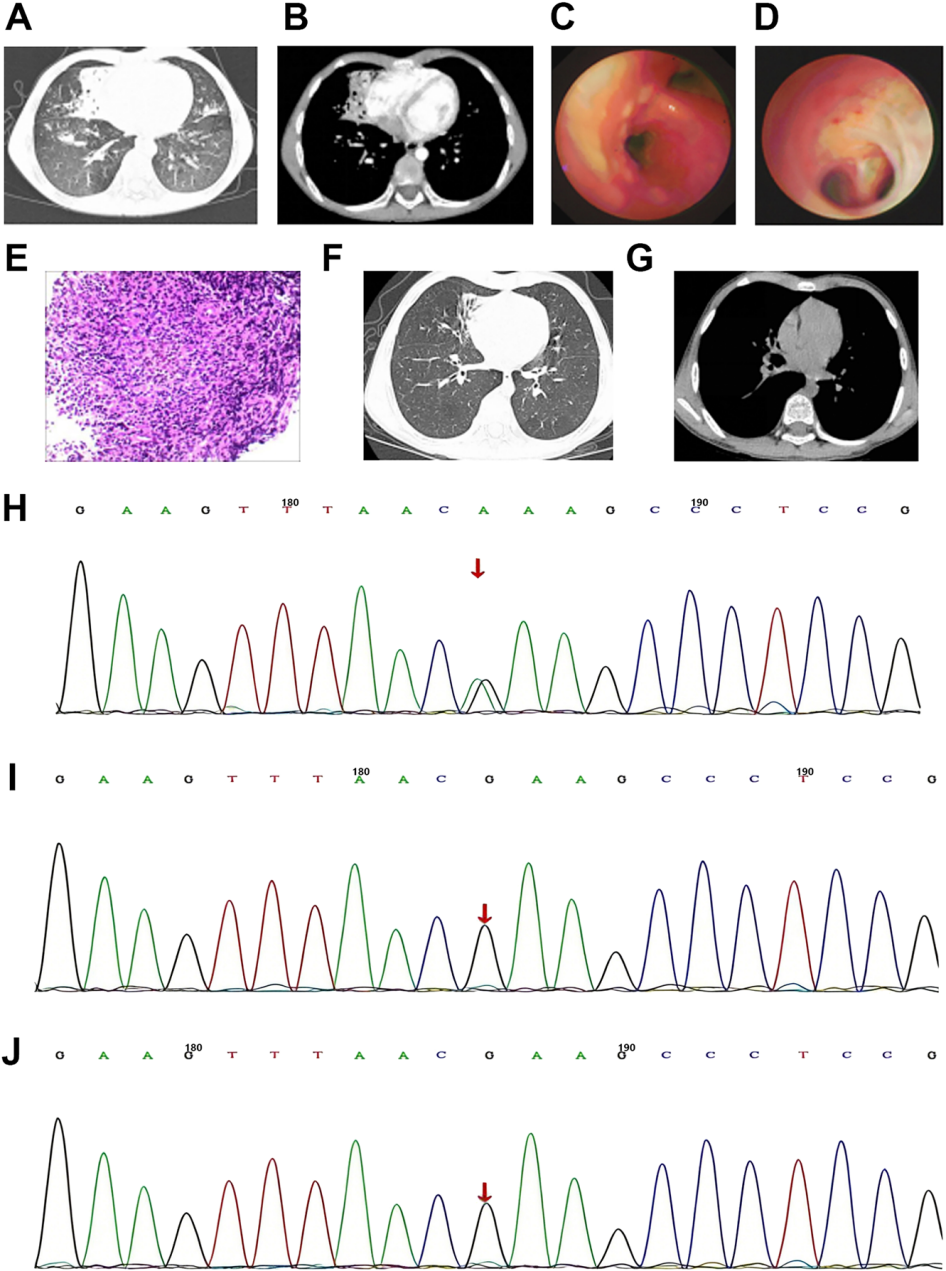
Examinations findings of case 3. (A) Initial Lung window on chest CT before treatment. (B) Initial mediastinal window of chest CT before treatment. (C) Bronchoscopy seen at Massive bronchial mucosal follicular hyperplasia before treatment. (D) Bronchoscopic view of distal airway bronchial dilatation before treatment. (E) Mucosal biopsy (HE × 40) lymphocytic infiltration after treatment. (F) Lung window on chest CT after 3-month follow-up of treatment. (G) Mediastinal window on chest CT after 3-month review of treatment. (H) The affected child PIK3CD heterozygous mutation in gene c.3061G > A (p.E1021K). (I) No mutation detected in the father of the affected child. (J) No mutation detected in the mother of the affected child.

Auxiliary examinations: Complete blood count: WBC 8.6 × 10^9^/L, lymphocyte percent: 18.30 %, hemoglobin 121.00 g/L, platelets 217 × 10^9^/L. Immunological parameters are shown in Table 1. EBV IgG was positive within the normal quantitative range. TB infection T-cell test and PPD test were negative. *M. pneumoniae* RNA was positive. High-throughput DNA sequencing of pathogenic microorganisms detected: *Haemophilus* spp. (263 sequences), *Haemophilus influenzae* (54 sequences), *Streptococcus* spp. (3 sequences), *S. pneumoniae* (2 sequences), human gammaherpesvirus 4 (9 sequences), and torque tenovirus 12 (3 sequences). Lymph node ultrasound showed bilateral cervical and axillary lymphadenopathy. Contrast-enhanced chest CT indicated sinusitis, bilateral otitis media, pneumonia with partial atelectasis and local ischemic changes in the right middle lobe, with no evidence of tuberculosis. Pulmonary function tests revealed mild obstructive ventilatory impairment (Figure 3A and B).

Treatment: After admission, anti-infective therapy with cefoperazone-sulbactam and linezolid was initiated, supplemented with oral itraconazole, isoniazid, and rifampicin. On hospital day 5, bronchoscopy (Figure 3C and D) showed tracheobronchitis with uneven mucosal surfaces suggestive of lymphoid hyperplasia, more pronounced on the right side. Mucosal biopsies and brushings were performed. The lumen contained flocculent secretions and appeared congested; the lateral wall of the right middle bronchus was externally compressed with associated bronchial dilation. Rapid on-site cytologic evaluation (ROSE) of bronchial brushings revealed numerous neutrophils, lymphocytes, and monocytes, with granulomas and multinucleated macrophages noted, consistent with an inflammatory/infectious process. Fungal or tuberculous infection could not be excluded, and acid-fast staining was suspiciously positive. Histopathological examination of the bronchial mucosa showed squamous hyperplasia with dense submucosal lymphocytic infiltration (Figure 3E). Electron microscopy of nasal and airway epithelia showed sparse, markedly degenerated cilia with unclear internal structures. The patient’s clinical symptoms improved after 3 weeks of treatment, and he was subsequently discharged.

Genetic testing identified the same heterozygous mutation in the PIK3CD gene as in Cases 1 and 2: c.3061G > A (p.Glu1021Lys, E1021K). This was confirmed as a *de novo* mutation, as it was not detected in either parent (Figure 3H–J) (Beijing MGI Medical Laboratory, sample number: 19C122314).

Follow-up: After discharge, he continued oral anti-tuberculosis drugs for 3 months, along with traditional Chinese medicine for immune modulation and nebulization therapy. Re-evaluation 3 months later showed a significant reduction in cough and sputum production. Chest CT revealed residual pneumonia and localized bronchiectasis in the right middle lobe (Figure 3F and G). Pulmonary function tests indicated mild small airway obstructive ventilatory impairment.

**Informed consent:** Informed consent has been obtained from all individuals included in this study.

**Ethical approval:** The research related to human use has been complied with all the relevant national regulations, institutional policies and in accordance with the tenets of the Helsinki Declaration, and has been approved by the Ethics Committee of Affiliated Children’s Hospital, Shandong University.

## 3 Discussion

APDS results from gain-of-function mutations in the PIK3CD gene. These mutations disrupt the inhibitory interaction between the p110δ catalytic subunit (encoded by PIK3CD) and the p85α regulatory subunit (encoded by PIK3R1) [5], leading to constitutive over-activation of the crucial PI3K-AKT-mTOR signaling pathway involved in cell proliferation and survival [6]. To date, 12 distinct heterozygous missense mutations have been confirmed as pathogenic. In this study, all three patients with APDS harbored the E1021K amino acid substitution resulting from the c.3061G > A variant. This mutation, located within the kinase domain of p110δ, is the most frequently reported worldwide. Wang et al. [7] further elucidated its pathogenicity through phenotypic analysis in a patient and a corresponding Pik3cdE1024K+/+ mouse model, confirming a homozygous mutation caused by maternal segmental uniparental disomy and a maternal chromosomal insertion.

The PI3K pathway plays a critical role in B-cell maturation, apoptosis, and lymphocyte migration [8]. Furthermore, PI3K and mTOR signaling regulates the expression of adhesion and chemokine receptors on T-lymphocytes, thereby influencing T-cell metabolism, activation, effector functions, and recirculation [9]. Dysregulation of this pathway consequently leads to a combined immunodeficiency. The typical immune phenotype of APDS includes reduced CD4+ T-cells, increased CD8+ T-cells, a low CD4/CD8 ratio, decreased B-cell counts (particularly naïve B-cells), and an expanded transitional B-cell population. Hypogammaglobulinemia (low IgG), low IgA, and elevated IgM are commonly observed [10]. In our case series, comprehensive lymphocyte immunophenotyping – such as detailed naïve/memory T- and B-cell subset analysis – was not performed as part of the initial clinical assessment. This limits a full characterization of the immune phenotype beyond the basic CD4+/CD8+ counts and immunoglobulin levels presented in Table 1. As shown, all three cases demonstrated the hallmark findings of decreased CD4+ T-cells, increased CD8+ T-cells, and a reduced CD4/CD8 ratio, which appeared to correlate with age or disease severity. Immunoglobulin profiles were variable: IgG was normal or slightly low in all patients. IgM was at the upper limit of normal or mildly elevated in Cases 1 and 3, while IgA levels were mostly normal or slightly decreased. Notably, Case 2 presented with markedly elevated IgM and significantly reduced IgA. This patient also exhibited more severe clinical symptoms and pronounced hepatosplenomegaly compared to Cases 1 and 3. This pattern suggests that the degree of IgM elevation and IgA deficiency may correlate with disease severity, a hypothesis that warrants further validation in larger cohort studies.

APDS has diverse clinical manifestations. In infants and young children, it often presents with recurrent respiratory tract infections, which can lead to bronchiectasis. The main clinical features are hepatosplenomegaly, CMV and/or EBV viremia, and lymphoid tissue hyperplasia. In addition, some children may experience recurrent or chronic diarrhea, purulent or bloody stools, inflammatory bowel disease, developmental delay, hematological changes, and some may eventually develop lymphoma or autoimmune diseases such as immune thrombocytopenia and arthritis. According to large-scale reports from abroad, the clinical manifestations of the disease vary greatly. Mild cases can lead to no symptoms in adults, while severe cases can present with severe immunodeficiency symptoms in early stages [11].

To contextualize our findings within the known spectrum of APDS, we compare our three cases with previously reported patients carrying the same E1021K mutation. The core phenotype observed in our cohort – recurrent sinopulmonary infections leading to bronchiectasis, lymphadenopathy, and the pathognomonic finding of NLH on bronchoscopy – closely aligns with the major diagnostic features described in larger series [11]. Immunologically, the trend of low CD4+/CD8+ ratio, decreased IgA, and elevated IgM, particularly evident in our more severe Case 2, also reflects the characteristic dysregulation reported in APDS [12]. However, our cases underscore the significant clinical heterogeneity even among patients with an identical genetic lesion. While hepatosplenomegaly is a common feature in APDS, its presence and severity varied markedly in our cohort: it was a prominent and progressive finding in Case 2 but was minimal or absent in Cases 1 and 3 at similar follow-up points. Furthermore, the onset and progression of structural lung disease differed; Case 2 had early and recurrent pneumonias, while bronchiectasis in Case 1 was a later development post-diagnosis. This spectrum of severity, from chronic otitis media and recurrent pneumonia to significant immune cytopenia (as seen in Case 3), highlights that the E1021K mutation does not dictate a uniform clinical course. The variability in treatment response – from significant improvement with rapamycin in Case 2 to a reliance on IVIG and antimicrobials in others – further emphasizes the need for individualized management strategies. These observations reinforce that PIK3CD-related APDS is a disease with a variable expressivity, where identical genotypes can manifest with divergent phenotypic severity.

In this series, the first and third cases developed chronic respiratory tract infections at approximately 3 years of age. In contrast, the second case exhibited recurrent respiratory infections from birth, which progressively worsened and were accompanied by chronic otitis media. Bronchiectasis was documented in all three patients; notably, it emerged in the first case two years after the initial diagnosis. Multiple lymphadenopathy was a consistent finding. Additionally, the second case presented with hepatosplenomegaly that manifested in the later disease stages, while the third case had concurrent immune thrombocytopenia. As APDS is a combined immunodeficiency, the spectrum of infectious pathogens is broad and non-specific, encompassing viruses, bacteria, and, less commonly, fungi and mycobacteria. *S. pneumoniae* is frequently identified as a bacterial culprit. Consistent with the literature, these patients demonstrated heightened susceptibility to viral infections such as EBV and CMV. Specifically, *S. pneumoniae* was isolated from bronchoalveolar lavage culture in the first case, who later had infections with Salmonella, EBV, cytomegalovirus (CMV), and adenovirus. The second case had a sputum culture positive for *Candida albicans*, and serology indicated infections with herpes simplex virus, coxsackievirus, adenovirus, and CMV. The third case was infected with *M. pneumoniae* and EBV, alongside a concurrent *Mycobacterium tuberculosis* infection. Common imaging features across all three cases included persistent lymphadenopathy, pulmonary consolidation, progressive bronchiectasis, and potential lung atelectasis.

The global incidence of APDS has not been well established due to its diverse clinical and immunological presentations; definitive diagnosis currently relies on genetic testing. With the advancement and increasing availability of genetic technologies, reported cases have accumulated gradually. However, the variable age of onset in pediatric patients poses a significant challenge for early clinical recognition. In our series, bronchoscopic examination in all three cases revealed an irregular mucosal surface with diffuse nodular lymphoid hyperplasia, consistent with the endoscopic findings reported in a case from Beijing Children’s Hospital [12]. Furthermore, bronchoscopic mucosal biopsies were obtained in all patients, and case 2 additionally underwent transbronchial lung biopsy (TBLB). Histopathological analysis of the mucosal biopsies demonstrated extensive lymphocytic infiltration in each case. These observations reinforce that nodular lymphoid hyperplasia of the respiratory (or gastrointestinal) mucosa represents a characteristic feature of APDS [13], providing an important diagnostic clue. Notably, in two of our cases, follow-up bronchoscopy showed a marked reduction or even complete resolution of the previously observed lymphoid follicles compared to the initial examination. This change may be attributable to thorough endoscopic lavage, extended anti-infective therapy, and the timing of the repeat procedure. Therefore, bronchoscopy not only allows for evaluation of mucosal changes and assessment of treatment response, but endoscopic lavage also helps clear mucous plugs, restore airway patency, promote lesion resolution, and identify pathogens before the development of distal bronchiectasis as seen on imaging. Bronchoscopy thus plays a valuable role in both the diagnosis and management of APDS and merits greater clinical attention.

The immune dysfunction caused by APDS is primarily mediated through the constitutive activation of the PI3Kδ-AKT-mTOR pathway, distinguishing it from other immunodeficiencies and offering a unique avenue for targeted therapy. Currently, several treatment strategies exist, each with distinct profiles. Rapamycin (sirolimus), an mTOR inhibitor, has demonstrated efficacy in mitigating lymphoproliferation and hepatosplenomegaly, as evidenced in our Case 2. However, its use is limited by potential pulmonary toxicity, immunosuppressive effects increasing infection risk, and practical challenges in sourcing and monitoring in pediatric populations, particularly in China. The PI3Kδ inhibitor idelalisib, used primarily in hematologic malignancies, is not approved for APDS due to significant adverse effects including fatal hepatotoxicity and intestinal perforation. Hematopoietic stem cell transplantation (HSCT) remains a potentially curative option. Successful outcomes have been reported, especially in patients with severe, refractory disease or impending organ damage [11]. However, HSCT carries substantial risks of regimen-related toxicity, graft-versus-host disease, and high procedure-related mortality. In our cohort, given the spectrum of disease severity – ranging from manageable with supportive care (Cases 1 & 3) to more progressive (Case 2) – the decision for HSCT would require stringent, individualized risk-benefit assessment. For patients like our Case 2 with significant and progressive hepatosplenomegaly, HSCT could be considered if symptoms become refractory to targeted medical therapy. The recent international retrospective analysis provides further valuable data on outcomes and factors influencing HSCT success in APDS, reinforcing the need for careful patient selection [14].

Recently, leniolisib, a highly selective PI3Kδ inhibitor, has emerged as a promising targeted therapy. Clinical trials have shown significant efficacy in reducing lymphoproliferation, improving immunophenotype, and normalizing IgM levels in APDS patients, with a favorable safety profile in the study period [15]. Based on its mechanism and reported efficacy, we would consider leniolisib a first-line targeted therapeutic option for our patients, particularly for those like Case 2 with prominent lymphoproliferation and humoral dysregulation. Its targeted action may offer a superior benefit-risk ratio compared to broader immunosuppressants like rapamycin. However, we must also consider different perspectives on long-term safety. While initial data are encouraging, PI3Kδ inhibition can theoretically alter immune surveillance. A perspective article cautions about the need for long-term vigilance regarding possible adverse events, including infections or unforeseen immune dysregulation [16]. Furthermore, foundational research highlights the critical role of PI3Kδ in immune function, underscoring the importance of sustained monitoring for any therapy modulating this pathway [17]. Therefore, our thesis is one of cautious optimism: leniolisib represents a major advance for precision therapy in APDS, but its initiation should be followed by meticulous long-term follow-up within registries or structured clinical protocols to fully define its safety profile.

In our specific cases, after diagnosis, all three children received regular intravenous immunoglobulin replacement. Only Case 2 received rapamycin with good response. Considering the current landscape, we would propose a stratified approach: For a new, similar patient today, we would prioritize enrollment in a leniolisib access program or clinical trial if available. HSCT would be reserved for patients with severe, life-threatening complications refractory to optimal medical management, including targeted inhibitors. This framework aligns with moving towards precision medicine while acknowledging the need for ongoing evaluation of novel therapies.

In summary, activated PI3K-δ Syndrome is a rare, combined primary immunodeficiency characterized by significant clinical heterogeneity and a high risk of underdiagnosis, with definitive diagnosis reliant on genetic testing. Although its incidence is low, the disease carries substantial morbidity, posing a serious threat to both survival and quality of life in affected children. Therefore, in pediatric patients with recurrent respiratory infections and abnormal chest imaging, clinicians should maintain a high index of suspicion, particularly when accompanied by aberrant immunological markers. Prompt bronchoscopy is recommended, as the finding of extensive nodular lymphoid hyperplasia provides a critical diagnostic clue. Suspected cases should undergo expedited genetic testing to enable early diagnosis and guide timely, targeted therapeutic intervention.
